# Assessing Occupational Safety Risks and Challenges Among Informal Welders in Pretoria West, South Africa

**DOI:** 10.3390/ijerph22071132

**Published:** 2025-07-17

**Authors:** Marvin Mashimbyi, Kgotatso Jeanet Seisa, Muelelwa Ramathuthu, Maasago Mercy Sepadi

**Affiliations:** Faculty of Science, Environmental Health, Tshwane University of Technology, Pretoria 0183, South Africa; mashimbyimarvin@gmail.com (M.M.); seisajeanet@gmail.com (K.J.S.); moelelwaramathuthu@gmail.com (M.R.)

**Keywords:** informal welders, informal economy, occupational safety, injury risk, PPE compliance, sustainable development goals, South Africa

## Abstract

Background: Informal welders in Pretoria West face growing occupational safety risks due to hazardous working environments and limited regulatory oversight. Despite the high-risk nature of their work, many remain unaware of relevant safety legislation and inconsistently use personal protective equipment (PPE). This study aimed to investigate the occupational safety risks, challenges, and levels of compliance with safety practices among informal welders in Pretoria West, South Africa. Methods: A cross-sectional mixed-methods approach was employed, incorporating both qualitative and quantitative designs. Data were collected from 40 male welders (aged 20–55 years) using structured questionnaires, observational checklists, and semi-structured interviews. Descriptive statistics were generated using Microsoft Excel, while thematic content analysis was applied to the qualitative data. Results: Eighty-five percent (85%) of welders reported using gas welding, and more than half had received training in welding and PPE use; however, 47.5% had no formal training. A high prevalence of work-related injuries was reported, including burns, cuts, and eye damage. Common safety concerns identified were burns (42.5%), electric shocks (35%), and malfunctioning equipment. Observational data revealed inconsistent PPE use, particularly with flame-resistant overalls and eye protection. Qualitative insights highlighted challenges such as demanding client expectations, hazardous physical environments, and inadequate equipment maintenance. Many sites lacked compliance with occupational safety standards. Conclusion: The study reveals critical gaps in safety knowledge, training, and PPE compliance among informal welders. These deficiencies significantly elevate the risk of occupational injuries. Strengthening occupational health and safety regulations, improving access to PPE, and delivering targeted training interventions are essential to safeguard the well-being of welders and those in their surrounding communities.

## 1. Introduction

Welding is a widely used fabrication technique, encompassing methods such as shielded metal arc welding and gas metal arc welding, and is commonly applied across sectors including construction, automotive, and manufacturing industries [1,2]. In many urban communities, particularly in developing countries, welding serves as a key source of employment and supports vital infrastructure development [3]. However, despite its economic contributions, welding remains a high-risk occupation. Globally, more than 200 million occupational accidents occur annually, with over 200,000 fatalities reported in developing countries, many of which are linked to hazardous welding environments [1,4].

In the African context, welding constitutes a core component of the informal economy. An estimated 80% of informal welding enterprises operate without formal oversight or regulation, exposing workers to multiple occupational health and safety risks. These include inadequate training, inconsistent or nonexistent use of personal protective equipment (PPE), and restricted access to occupational healthcare services [1,4,5]. Informal workers typically operate in settings characterized by precarious income, limited infrastructure, and weak institutional enforcement. As a result, they are disproportionately vulnerable to work-related injuries and long-term health effects.

Despite these well-documented risks, many informal welding operators remain unaware of key regulatory frameworks such as the Occupational Health and Safety Act No. 85 of 1993, which mandates the implementation of workplace safety policies and enforcement mechanisms [6]. The absence of regulatory enforcement not only endangers welders but also poses broader public safety risks to surrounding communities [7].

Empirical studies conducted in similar low- and middle-income countries (LMICs) have reported alarmingly low levels of PPE use and hazard awareness among informal welders. For example, a cross-sectional study in Nepal found that less than half of welders consistently used basic safety equipment, with many citing cost and discomfort as primary barriers [8]. Similarly, research in South Africa has linked informal welding work to a high prevalence of musculoskeletal disorders and exposure-related respiratory risks, compounded by poor ergonomic design and environmental hazards [9]. These findings underscore the urgent need for localized, context-specific investigations into occupational risks in informal welding environments.

While existing research has examined general occupational hazards in informal sectors [2,3,5], few studies focus specifically on the unique dangers faced by informal welders in South Africa. Much of the literature either generalizes across informal occupations or neglects the structural, economic, and behavioral dimensions that shape safety outcomes in welding. This represents a critical knowledge gap, particularly given the frequency of injuries, the lack of hazard recognition, and the minimal regulatory protection afforded to informal workers.

This study seeks to fill that gap by examining safety practices, injury patterns, and risk awareness among informal welders in Pretoria West. Employing a mixed-methods approach, it integrates quantitative data on injury frequency and PPE compliance with qualitative interview narratives and structured site observations. This methodology allows for a context-sensitive and comprehensive understanding of real-world safety behaviors in informal settings. Notably, the use of cluster analysis to segment behavioral profiles of welders offers a novel methodological contribution, facilitating the design of targeted, evidence-informed safety interventions. The findings aim to inform both occupational health policy and grassroots-level safety initiatives to improve the well-being of informal workers in South Africa. Moreover, the study aligns with the Sustainable Development Goals, particularly SDG 8, which promotes “decent work for all” and inclusive, sustained economic growth.

### 1.1. Study Focus

This study centers on informal welding businesses operating in Pretoria West, where regulatory oversight and occupational infrastructure are minimal. These enterprises often function with limited resources, exposing workers to serious health and safety risks. The study aims to investigate the occupational safety risks and challenges faced by welders in these informal settings. Specifically, the research explores worker demographics, PPE knowledge and usage, injury history, safety compliance, and awareness of health and safety regulations [2,8]. The findings aim to support policy development and practical interventions that can reduce occupational hazards, protect the well-being of informal welders, and foster more sustainable industrial development within the informal economy.

### 1.2. Research Questions

To achieve the study’s objectives, the following research questions were addressed:

(1) What are the common occupational risks and injuries experienced by informal welders in Pretoria West?
(2) To what extent do informal welders have access to and use personal protective equipment (PPE)?
(3) What level of awareness do informal welders have regarding occupational health and safety regulations?
(4) What environmental or operational challenges do welders face in informal workplaces?
(5) How compliant are informal welding sites with existing occupational safety standards?

## 2. Materials and Methods

The study employed a cross-sectional design using a mixed-methods approach to investigate the occupational safety risks and challenges faced by informal welders in Pretoria West.

### 2.1. Study Area

A mixed-methods approach was used to combine both quantitative and qualitative study designs [9,10] to collect data. The quantitative component involved the administration of structured questionnaires, while the qualitative component involved in-depth interviews with the welders, using a semi-structured interview guide to obtain more detailed information from expressed experiences, opinions, and challenges of the welders [11]. This mixed-methods approach allowed for triangulation of findings, by providing a more comprehensive understanding of the research phenomenon [12].

### 2.2. Population & Setting

The target population of this study comprises all welders operating as informal welders at unauthorized workplaces in Pretoria West, which is a rapidly industrialised area in the City of Tshwane Metropolitan Municipality, Gauteng Province, South Africa, with a total population of about 110,000+ (from the 2022 census) [13]. According to Statistics South Africa [13], there are approximately 28 metal fabrication businesses in the area.

### 2.3. Sampling Methods

A sample size of 40 welders was recruited from different welding workplaces in Pretoria West. To ensure that welders had meaningful working experience, the study focused on welders with a minimum of six months of work experience to guarantee that respondents (welders) would provide informed perspectives on workplace safety practices and challenges in informal welding environments. Sampling techniques [14], non-probability sampling was used to select a representative sample of welding workplaces.

Within these workplaces, convenience and snowball sampling techniques were used to recruit individual welders who met the inclusion criteria, whereby we approached welders who were present, easily accessible, and willing to participate during data collection. All 40 selected participants completed the questionnaires and interviews.

The sample size of 40 participants (100%) was methodologically appropriate for the study’s cross-sectional mixed-methods design, particularly given the exploratory nature of the research and the depth of data required from informal settings. In qualitative and community-based studies, smaller sample sizes are often sufficient to generate meaningful insights when participants are purposefully selected to represent a range of experiences and conditions. The size was also influenced by practical considerations, including the challenges of accessing informal welders, the need for diversity in workplace settings, and the absence of external funding, as the research was conducted in partial fulfillment of a degree qualification. Given these constraints, a sample of 40 was both feasible and adequate for achieving the study’s objectives.

### 2.4. Data Collection Tools and Procedures

Data was collected using questionnaires, an observational checklist, and semi-structured interviews. All 40 (100%) participants were assessed using both a questionnaire and interview questions. Each participant will answer both tools, then use the checklist to assess their workplace.

#### 2.4.1. Observational Checklist

An observational checklist was used to evaluate the welding workshops. This tool has facilitated the identification of potential safety risks and hazards present in the workplace, the assessment of the availability of safety and emergency measures, and the general condition of safety equipment, as we did a walkthrough and inspected the workplaces in which welders were operating.

#### 2.4.2. Welder Questionnaire and Interviews

A structured questionnaire was administered face-to-face with participating welders during their working hours to collect quantitative data. The questionnaire was designed through a comprehensive review of relevant literature, consultation with occupational health professionals, and adaptation of previously validated tools from welding safety research. It was structured to collect detailed information on participants’ demographic profiles, occupational characteristics (such as welding techniques used, work schedules, and use of personal protective equipment), personal habits (e.g., smoking), experiences with work-related injuries, barriers to accessing healthcare and reporting incidents, as well as welders’ comfort in communicating health concerns with their employers. The questionnaire was used to conduct the assessment of safety awareness among welders, to assess the knowledge of workplace hazards, PPE requirements, and access and rights to the Occupational Health and Safety Act. The questionnaires were administered through researcher-assisted interviews to accommodate participants with limited literacy or comprehension difficulties. This approach yielded positive engagement, with many respondents reporting enhanced understanding of the survey items, while others expressed appreciation for the guided administration process. The questionnaire was pilot tested with 10 welders to ensure the clarity, validity, and relevancy of the questions. During data collection, those who were able to complete the questionnaire on their own self-administered it, and those who could not complete it (by means of writing or reading) were administered by the researcher, based on what they were responding.

Individual semi-structured interviews were conducted to gather in-depth qualitative data on welders’ challenges, experiences, and opinions related to occupational safety, and the interview explored difficult welding experiences encountered by the welders; specific safety concerns; fears that welders would have when they are welding; perceived barriers to implementing safety measures; and experiences with physical health issues, which are potentially related to their work [15]. During the data collection phase, the researchers conducted face-to-face interviews by posing questions directly to participants and recording their responses. To ensure the validity and contextual appropriateness of the interview tool, the questions were pilot tested with 10 informal welders from a similar setting prior to implementation. This process allowed for refinement of question wording and structure to enhance clarity and relevance for the target study population.

##### Core Questions from the Survey and Interview Instruments

The questionnaire and interview guides were structured around core research questions aimed at assessing training, safety behavior, injury experience, and risk awareness. Examples of key questions included: Have you received any formal or informal training for welding? Do you regularly use personal protective equipment (PPE) when you work? Have you ever experienced work-related injuries or accidents while welding? And are you aware of any specific safety risks in your workplace?

### 2.5. Inclusion and Criteria

The study included participants who were currently working as informal welders in Pretoria, at least 18 years of age, with a minimum of 6 months of welding experience, willing to participate, and able to understand and complete the questionnaire, and those who had provided informed consent for participation. The study has excluded informal welders who were under 18 years of age, had less than 6 months of experience, non-welding employees, and those unable to provide informed consent.

### 2.6. Data Analysis

The data collected was captured using an Excel spreadsheet and analyzed through Microsoft Excel 2016 [16,17].

- Quantitative data collected through questionnaires and observational checklists were analyzed using descriptive statistics, and quantitative data using data visualization [18]. Specifically, descriptive statistics were used to summarize demographic information, safety risk perceptions from the participants, and their opinion responses [19]. This data was used to identify relationships between variables, such as the impact of training programs on safety knowledge and behavior [5]. The quantitative data collected was analyzed through graphs and tables.
- Qualitative data were obtained through semi-structured interviews and analyzed using thematic analysis [20]. Analysis followed Braun and Clarke’s (2006) six-phase framework [20,21]. The interview transcripts were read thoroughly by J Seisa and M Mashimbyi, two researchers of this study, to achieve familiarization with the content from the responses. Open coding was then conducted independently by the same researchers to identify initial codes. We then met to discuss similarities and differences in coding and refine codes, following a step of grouping the codes into potential themes. These themes were reviewed, interpreted, and refined through a repetition process, ensuring that they accurately reflect the data. Lastly, we defined the themes, and coding and sub-coding were selected to support the findings. To ensure the reliability of the qualitative analysis, we have assessed inter-coder reliability, where Cohen’s kappa coefficient [22] was calculated to measure the agreement between the researchers’ coding. A Kappa value of 0.82, which indicates strong agreement, confirms the reliability of the coding process. The results analyzed and presented ought to protect the autonomy of the participants and not reveal their identity [23].

## 3. Results

### 3.1. Demographic Profile of Participants

Table 1 below illustrates the demographic and occupational information of the participants included in the study conducted in Pretoria West, with all 40 (100%) of the participants being male. The ages of the participants included in the study ranged from 20 to 55 years old, as shown in Table 2. Among the 40 participants, most preferred gas welding, and only a few preferred Arc and MIG welding.

#### Welders’ Work Schedule

**Figure 1:**
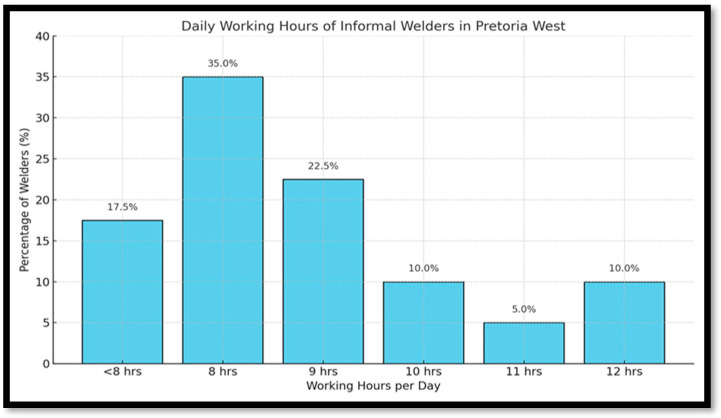
Bar graph, participants’ work schedule (in hours).

Figure 1 shows the participants included in the study, working on a flexible schedule. The data shows their daily working periods, providing a comprehensive overview of their daily working routines. Most participants in the study, 13 (35%), worked for 8 h daily. However, the most frequent hours were 9 h daily, 9 (22.5%), and 10 h daily, 4 (10%). Another small number of participants worked long hours, such as 12 h daily, 4 (10), and 11 h daily, 2 (5%). A few, 7 (17.5%), participants were working less than 8 h a day. Most participants (welders) took 1 h breaks, followed by those taking 30 min breaks, and those who worked daily took breaks depending on the availability of clients; 2 and 3 h breaks were the least, with only a small percentage of participants taking them.

### 3.2. Theme 1: Awareness of Safety Hazards and Risks in Welding Workplaces

Most participants reported being burned by sparks, molten slag, and flames while welding. Statements from participants, such as “I was burnt by flames when welding connecting two metals,” reveal frequent contact with high-temperature materials and sources of flames, such as a grinder. The burns affected various parts of the participants, including the hands, arms, and legs. Several responses stated that they suffer from injuries (cuts) from grinders, and electric shocks from welding machines, with comments like “I got cut by a grinder” and “cutting by the welding machine”. These types of injuries can be prevented by using welding tool handling. Some participants mentioned having eye problems, which can be linked to exposure to sparks from welding machines, with some statements from participants, such as “Fumes getting into my eyes, when I’m not wearing googles”, “With fumes, and sparks getting inside my eyes”. This suggests that some workers may not be using safety goggles while welding.

#### Commonly Expressed Fears and Priorities: Word Cloud Analysis

**Figure 2:**
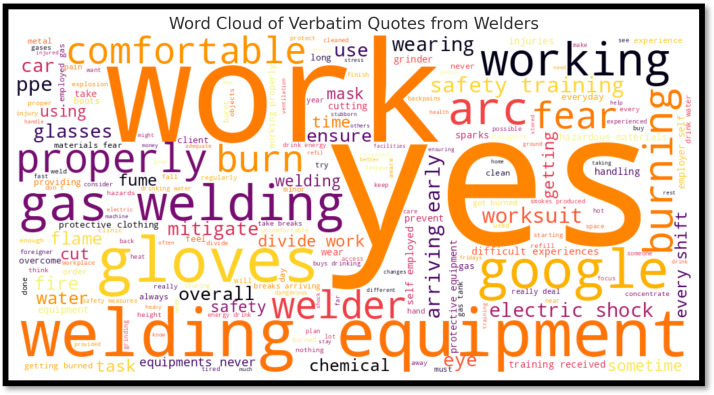
Word cloud of verbatim quotes from welders.

To capture welders’ most pressing safety concerns in their own words, a word cloud (Figure 2) was generated from the transcribed interview responses. This visualization highlights the most frequently mentioned terms across all qualitative data, providing insight into both emotional and operational dimensions of welding work in informal settings. The most dominant words in the cloud were “work,” “yes,” “equipment,” “gloves,” “gas,” “fear,” “burn,” and “welding,” which demonstrate that welders are highly attuned to the physical risks associated with their tasks. Frequent mentions of “gloves,” “goggles,” “mask,” “burn,” and “cut” indicate that personal safety, particularly regarding hand, eye, and respiratory protection, is a core concern. The term “fear” itself appears prominently, reflecting the emotional toll that repeated exposure to hazards can have.

Moreover, the appearance of words such as “training,” “safety,” “ensure,” and “mitigate” suggests that welders recognize the value of formal instruction and are open to strategies for reducing risk. However, the repeated use of simple affirmatives like “yes” may also reflect a limitation in how some welders express their experiences, potentially tied to literacy challenges or language barriers common in informal labor sectors. Together, these findings underscore that informal welders not only face real and ongoing safety hazards but also experience a degree of anxiety and uncertainty in managing them. These perceptions support the urgent need for empathetic, accessible safety training, context-sensitive communication, and consistent availability of PPE and guidance at the site level.

### 3.3. Theme 2: Access to Training and Safety Information

Further shows the training and experience of the participants in welding. Twenty-two (55%) participants reported receiving formal training in welding, while 18 welders stated they had no training. Twenty-one (52.5%) welders received training on protective equipment, whereas 19 (47.5%) welders did not. In addition to their occupational information, it examines the personal habits of the participants. Among the welders, 11 (27.5%) individuals reported being smokers, 28 (70%) reported being non-smokers, and 1 (2.5%) stated that their smoking status was unknown.

### 3.4. Theme 3: Perceived Causes of Injuries

#### 3.4.1. Welder’s Safety Concerns

**Figure 3:**
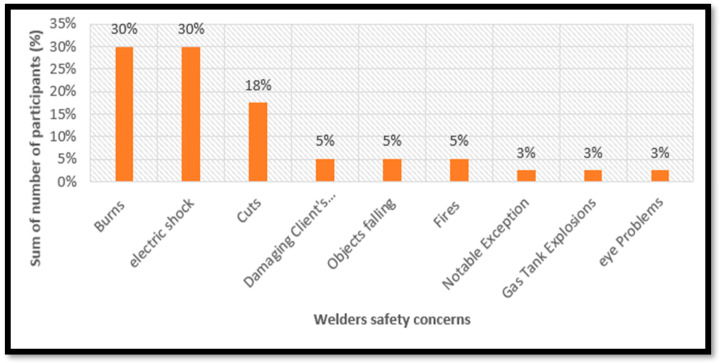
A graph showing safety concerns that welders fear encountering while welding.

About 40 welders in Pretoria West were asked to identify the challenges and risks they face in their occupation. The welders we interviewed expressed significant apprehensions about their work hazards and potential harm. Analysis of the responses to the question, “Are there any specific concerns or fears you have while welding?” has revealed two primary themes outlining the welder’s apprehensions. Figure 3 illustrates detailed analyses of those safety concerns that welders have. Almost all the welders (87%) expressed fear of getting hurt while working from work-related injuries such as burns, electric shock, cuts, fire, eye, and gas tank explosion. A smaller group of welders (10%) expressed concerns about damaging objects during their work, such as damaging their clients’ properties and objects falling from high areas. Notable exceptions were one welder (3%) reported having no specific safety concerns or fears.

The analysis of welders’ concerns in Pretoria West reveals a stark reality: many are deeply worried about their safety on the job. The top concerns are about getting hurt, especially from burns and electric shocks, which shows that welders are aware of the risks involved in welding. Some also worry about cuts, fires, eye problems, and explosions, although these concerns are less common. A smaller group is concerned about damaging objects while working.

#### 3.4.2. Challenges and Experiences Faced by Welders in Pretoria West

This section presents the key challenges experienced by welders in Pretoria West, based on semi-structured interviews conducted with 40 participants. The interviews explored one of the core questions: “What was one of your most difficult welding experiences?” The goal was to identify and categorize these difficulties to gain a clearer understanding of the real-world occupational hazards faced by informal welders. Approximately 25% of participants stated they had not encountered any particular welding challenges or could not recall specific incidents. Many of these welders attributed this to experience, routine, or careful task planning. For example, a Welder, aged 37 years, said, “I don’t find it difficult anymore because I know how to plan before I start.”

##### Client Pressure and Workload Strain (15%)

About 15% of welders reported difficulties in dealing with client expectations and managing multiple jobs simultaneously. They described scenarios where they felt overwhelmed by deadlines or confused by competing demands. A male welder aged 30, said, “It’s hard to focus when three different people are shouting about their jobs. You get confused, and that’s when mistakes happen.” (Male welder, age 30). These pressures can lead to reduced focus, rushed procedures, and an increased likelihood of error or injury.

##### Hazardous Work Environments and Complex Tasks (27.5%)

A significant 27.5% of participants shared experiences involving dangerous work conditions or technically challenging tasks. These include welding on elevated surfaces, working with deteriorated machinery, or joining incompatible metals. Some participants mentioned physically risky situations that were visually confirmed during site observations. One indicated that “Sometimes we weld things hanging from the roof… we must lie underneath. It’s scary because something can fall on you.” (Welder, age 34). While another indicated, “There are days I have to weld inside small spaces with no proper light. It’s risky, but we do it.” (Male, age 41). These challenges not only increase the risk of mechanical failure or accidents but also reflect a lack of standardized procedures in informal workshops.

##### Physical Injuries and Health-Related Complaints (27.5%)

About 27.5% of welders reported challenges stemming from physical injuries or discomfort. These included burns and cuts due to lack of PPE, discomfort from ill-fitting or low-quality protective gear, and symptoms such as dizziness, headaches, and chronic back pain likely linked to posture or prolonged standing. One welder stated, “I burned my fingers because I didn’t have proper gloves, only plastic ones.” (Male, 32). Another one indicated that “The mask makes it hard to breathe, but I wear it anyway.” (Welder, age 28). Another one stated, “My back is always painful after long hours standing in one place.” (Welder, 46). Such recurring injuries and ailments point to systemic safety gaps and inadequate ergonomic design in their work environments.

##### Equipment-Related Hazards (5%)

Approximately 5% of welders cited dangers associated with equipment failure or malfunction. Welders noted that aging or unreliable welding tools can cause electrical sparks, burnouts, or erratic behavior, creating hazards for both the user and those nearby. Welders stated that “Sometimes the machine sparks too much or stops halfway, and that’s when you can get shocked or burned.” (Male, age 39) and that “We borrow machines from each other, some are old and not safe, but we manage.” (Young welder, age 25).

##### Economic Barriers Affecting Safety Choices

Though not always explicitly stated as a “challenge,” many welders subtly described how the cost and availability of safety equipment influenced risky behavior. Welders stated, “Sometimes we don’t wear gloves because they are expensive.” (Male welder, age 45) and also said, “I share gloves with someone else. If he is using them, I just go without.” (Young welder, age 25). These statements highlight the economic constraints that often lead to non-compliance with safety practices, not due to ignorance, but due to limited access.

These issues can also affect productivity and job quality, especially when delays are caused by the need to replace or fix tools mid-task. These findings of welders’ most difficult experiences in Pretoria reveal a diversity of difficulties they face, the experiences show a demand of the nature of welding work and further highlight key factors that the majority of welders in Pretoria West experience physical injuries and must deal with complex or hazardous work environments, while others have never experienced any challenges.

#### 3.4.3. Barriers to Healthcare Reporting and Comfort in Discussing Health Concerns with Employers

Participants were asked about their experiences with healthcare and reporting work-related issues to help us understand their challenges concerning their safety concerns and occupational risks within their work environment. These aspects are important in understanding the occupational safety and health landscape for this population. We have identified barriers (see Table 3, which shows vulnerabilities in accessing necessary support, while comfort in communicating with employers influences proactive health management and reporting workplace incidents).

It was found in the analysis of the 40 participants’ responses, whereby most of the welders expressed that they have encountered no barriers in reporting injuries to their employers, some expressed that since they are foreigners, they lack documents that are required to access health facilities. They also expressed that the costs also restrict them from seeking medical attention, and considering the distance that they have to travel to these facilities, in their cases, they cannot afford to take time off from doing their work. Some welders responded that they have encountered barriers, but did not provide details.

Most welders have reported that they feel uncomfortable discussing health concerns with their employers, while others indicated they feel comfortable, and some have expressed that they are self-employed and do not have an employer to discuss health concerns with.

### 3.5. Theme 4: Usage and Compliance of Personal Protective Equipment (PPE)

**Figure 4:**
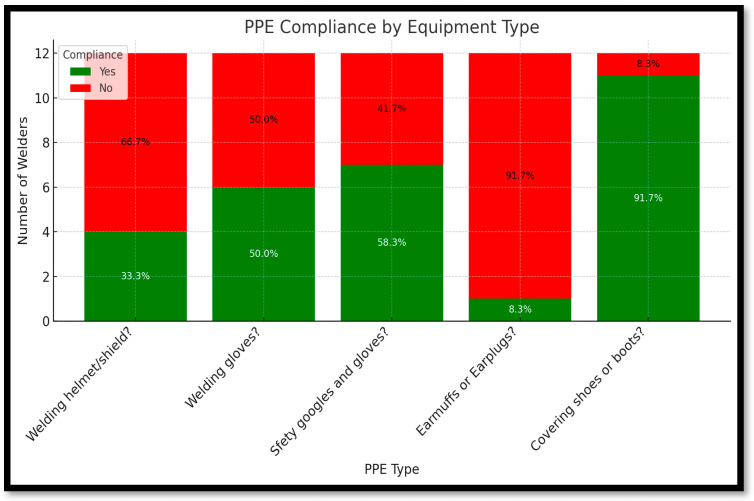
Showing different protective equipment usage (in percentage), by welders.

Figure 4 presents welders’ compliance with various types of personal protective equipment (PPE) based on observational checklist data. The graph displays both the percentage and the actual number of welders who reported using or not using specific PPE items. Among all equipment types, protective shoes or boots showed the highest level of compliance, with 11 welders (91.7%) consistently wearing them, and only 1 welder (8.3%) not using them. Similarly, safety goggles were used by seven welders (58.3%), with five (41.7%) not using them. Welding gloves reflected a balanced usage pattern, with six welders (50.0%) reporting compliance and the remaining six (50.0%) not using them. In contrast, earmuffs or earplugs recorded the lowest rate of usage, with just 1 welder (8.3%) using them and 11 welders (91.7%) reporting non-use. Flame-resistant overalls also had low compliance, worn by only three welders (25.0%), while nine (75.0%) did not use them. Welding helmets or shields were used by four welders (33.3%), and eight (66.7%) did not wear them during operations.

This distribution reveals clear patterns: while basic PPE like gloves and footwear is more widely adopted, protective gear for the head, eyes, and ears is consistently underutilized. These gaps are especially concerning given the types of hazards reported by welders, such as exposure to sparks, hot metals, loud noise, and flying debris. The results emphasize the need for targeted PPE education, better access to underused safety gear, and regular reinforcement of the importance of full-body protection in informal welding environments.

### 3.6. Frequency and Impact of Work-Related Injuries

**Figure 5:**
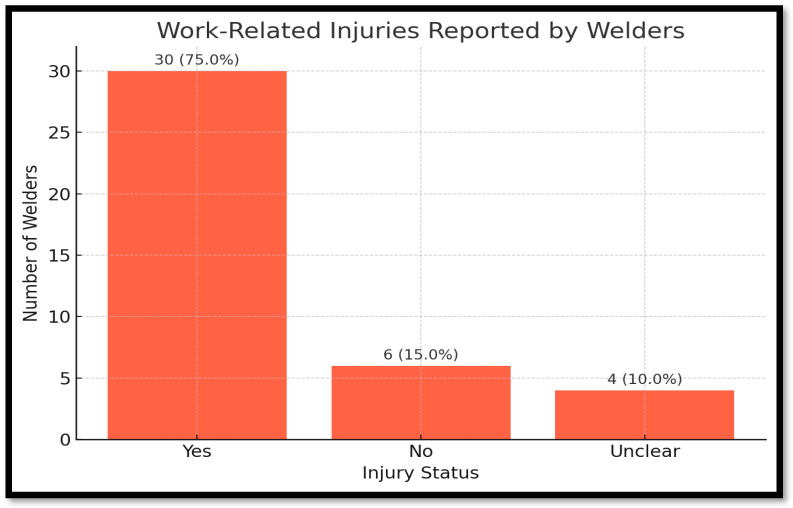
Injury frequency among welders.

Figure 5 shows a total of 30 welders (75.0%) reported having experienced a work-related injury, compared to only 6 welders (15.0%) who had not. An additional four respondents (10.0%) provided unclear or ambiguous answers. The high incidence of reported injuries aligns with the observed gaps in PPE compliance and site-level safety compliance discussed earlier. These injuries likely include burns from sparks, exposure to metal fumes, cuts from equipment, and eye damage, which are common risks in unregulated welding environments.

This sharp disparity between injured and non-injured welders provides strong evidence that safety interventions such as increased PPE usage, routine training, and regular site inspections are not only necessary but urgent. The data validates qualitative accounts from interviews and checklists and highlights that informal welders are operating in high-risk conditions without sufficient protection.

#### 3.6.1. Injury Frequency by Age Group

**Figure 6:**
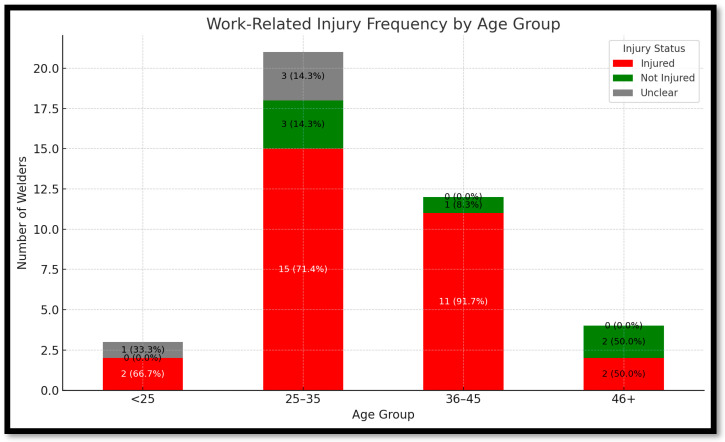
Work-related injury frequency by age group.

Figure 6 reveals a clear relationship between age and the likelihood of experiencing a work-related injury. Among the 25–35 age group, nine welders (69.2%) reported being injured, while four (30.8%) did not. The 36–45 group exhibited an even higher injury rate, with 11 welders (84.6%) injured and just 2 (15.4%) not. Interestingly, the youngest group, under 25, had a slightly better profile: four welders (50.0%) reported injuries, and the remaining four (50.0%) did not. In the 46+ group, the trend was less conclusive due to smaller numbers, but still showed six injured (75.0%) and two not injured (25.0%). These findings suggest that middle-aged welders (especially those between 36 and 45) may be at greater risk of occupational injury, potentially due to longer exposure, higher workload, or complacency in PPE use. Conversely, the youngest group may benefit from recent training or more cautious behavior. These trends reinforce the importance of targeted safety interventions based on age dynamics, particularly focusing on mid-career welders.

#### 3.6.2. Injury Frequency by Type of Welding

**Figure 7:**
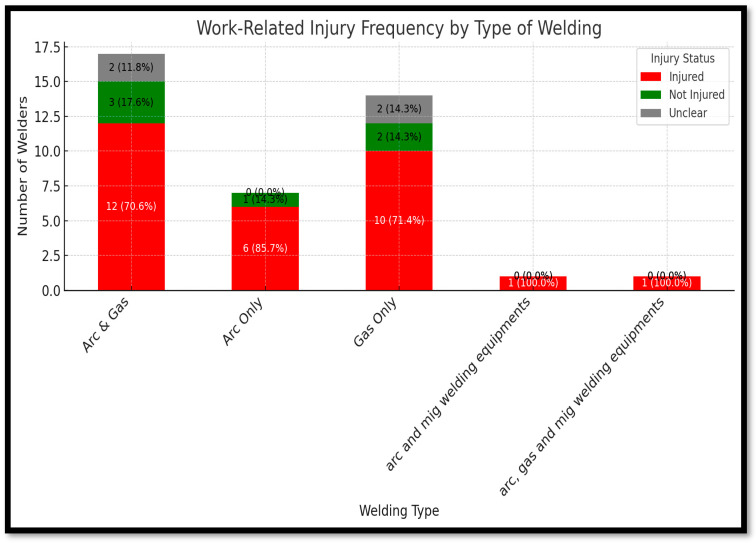
Work-related injury by type of welding.

Figure 7 shows the work-related injuries based on the type of welding equipment used, revealing notable differences in risk exposure. Welders using a combination of arc and gas welding equipment experienced the highest injury frequency, with 19 out of 26 (73.1%) reporting injuries, while 7 (26.9%) reported no injuries. Among those using arc welding only, three out of five (60.0%) reported injuries and two (40.0%) did not. In contrast, the gas-only group showed a lower injury rate, with three welders (42.9%) reporting injuries and four (57.1%) not. Welders using MIG welding reported no injuries; however, this subgroup consisted of only two participants, limiting generalizability. Additionally, five participants fell into an unclear category due to missing or ambiguous data on welding type or injury status. The results suggest that injury risk is elevated among welders who use multiple equipment types, particularly those who alternate between arc and gas welding. This may be due to the increased complexity, higher exposure to multiple hazards, or inconsistent application of safety practices across different techniques. Gas-only welders had comparatively lower injury rates, possibly because of more stable workflows or fewer moving parts. The absence of reported injuries among MIG welders is noteworthy but inconclusive due to the small sample size.

### 3.7. Site-Level Safety Compliance: Heatmap Analysis

**Figure 8:**
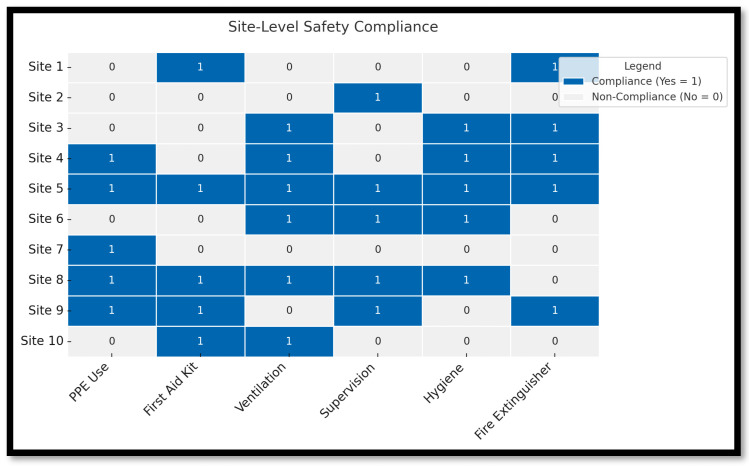
Heatmap of site-level safety compliance.

Figure 8 is a heatmap that visualizes the safety compliance levels across multiple informal welding workplaces observed in Pretoria West. Each row in the heatmap corresponds to a distinct welding site (not individual participants), while each column represents a specific safety compliance measure, such as the use of personal protective equipment (PPE), availability of first aid kits, ventilation, supervision, and hygiene practices. The color intensity in each cell reflects whether a particular safety measure was in place at a given site, where darker shades (closer to 1.0) represent full compliance (“Yes”) and lighter shades (closer to 0.0) indicate non-compliance (“No”). It is important to clarify that the site numbers in the heatmap are derived from physical workplace observations, not individual welder responses, ensuring that the analysis accurately reflects safety conditions at the environmental or institutional level.

The heatmap reveals significant variability in safety compliance among the welding sites assessed. While a few sites demonstrate strong adherence to basic protective measures, such as consistent use of welding helmets and gloves. Many others fall short in critical areas like emergency preparedness, record keeping, regular inspections, and hygiene practices. Notably, compliance with structured safety training sessions and equipment supervision was particularly weak across most locations, highlighting a widespread gap in occupational health infrastructure. These findings suggest that while some informal workshops make efforts to address PPE use, systemic safety planning, and regulatory oversight are largely absent. This uneven compliance pattern emphasizes the urgent need for targeted interventions, including training, site-level inspections, and the provision of standardized safety resources, to mitigate occupational hazards in these informal work settings.

### 3.8. Cluster Analysis of Welders’ Safety Behaviors

**Figure 9:**
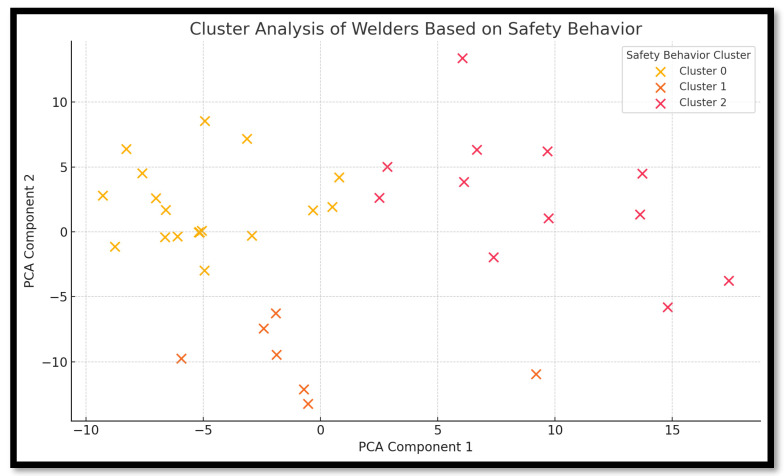
Cluster analysis of welders based on safety behavior.

To better understand variations in safety practices among informal welders, a cluster analysis (Figure 9) was performed using questionnaire responses related to training, PPE usage, injury experiences, and awareness of workplace risks. Using K-means clustering and principal component analysis (PCA), welders were grouped into three distinct behavioral profiles. Cluster 0 represents the Highly Compliant group of welders who received training, consistently use PPE, and are aware of occupational hazards. Cluster 1, the Moderately Aware group, includes welders who are somewhat compliant, possess limited training, and exhibit partial PPE use. Lastly, Cluster 2, the At-Risk group, consists of welders lacking formal training, PPE usage, and risk awareness, with a higher likelihood of reporting injuries or barriers to safe practices. This segmentation highlights the diversity of safety behavior among welders and underscores the need for tailored safety interventions, particularly for those in the “At Risk” group.

The cluster analysis underscores a striking disparity in how informal welders in Pretoria West approach occupational safety. The Highly Compliant group (Cluster 0) demonstrates characteristics of welders who may have access to better resources, mentorship, or a more structured work environment despite operating informally. These individuals are aware of the risks, take precautions, and likely have either formal or peer-guided training. Their safety-oriented behavior suggests that informal work settings can still cultivate compliance when basic infrastructure or community norms support it.

The Moderately Aware group (Cluster 1) reflects welders who are perhaps constrained by partial access to safety resources. They show some awareness and willingness to engage in protective behaviors but do not consistently follow through, often due to discomfort with PPE or inadequate training. This group represents a transition zone, where targeted interventions such as refresher training, improved access to PPE, and motivational programs could shift them toward full compliance. Their partial engagement suggests that behavior change is feasible with supportive strategies.

The At-Risk group (Cluster 2) raises the greatest concern. These welders are systematically deprived of training, neglect PPE, and are often unaware of occupational hazards, yet they continue to operate daily in high-risk environments. This group is not only vulnerable to physical harm but may also unintentionally contribute to unsafe practices that affect coworkers and the surrounding community. Their presence points to the urgent need for outreach, regulatory enforcement, and mobile training programs. Moreover, their clustering indicates that safety negligence is not random but concentrated, likely tied to specific workplace conditions, lack of policy oversight, or economic pressures.

These behavioral clusters highlight the need for differentiated safety interventions: Cluster 0 may benefit from reinforcement and recognition-based programs (e.g., model-site certification). Cluster 1 requires practical tools like subsidized PPE kits, hands-on demonstrations, and site-based coaching. Cluster 2 needs urgent attention via outreach by health authorities, fast-track training, and possibly stricter surveillance of high-risk workshops.

## 4. Discussion

### 4.1. Occupational Safety Risks and Site-Level Variability

This study highlights that informal welders in Pretoria West face considerable occupational risks due to inadequate training, inconsistent PPE usage, and structurally unsafe work environments. These findings align with literature identifying welding as a high-risk occupation globally, particularly in informal and unregulated contexts [24].

Among the 40 participants, the most commonly reported injuries were burns (25%), followed by eye injuries and lacerations. These were frequently attributed to inconsistent PPE use and a lack of formal training. Observational data confirmed that many welding sites were unregulated, lacked basic safety infrastructure (e.g., ventilation, fire extinguishers, first aid kits), and were located in high-traffic or open-air spaces without proper safety signage or supervision.

Previous research across sub-Saharan Africa corroborates these results. For example, in Uganda and Tanzania, informal welders reported high injury rates, particularly eye damage and burns, often due to a lack of goggles and gloves [25,26,27,28]. In the South African context, the Department of Labour’s Regulations for Hazardous Work on Machinery and the South African Bureau of Standards’ SANS 10238:2009 guidelines on Health and Safety in Welding and Allied Processes emphasize the mandatory use of protective gear such as goggles and gloves, yet enforcement in informal sectors remains weak [29,30]. Broader international guidelines, such as those by the International Labour Organization, similarly stress the importance of occupational health and safety measures, particularly for hazardous trades like welding [31]. Despite these frameworks, studies in Ethiopia, Uganda, and Nigeria found that most informal workers operated with substandard equipment and limited hazard awareness [27,32,33,34]. In many settings, workspaces lacked fire safety measures, and PPE was either unavailable or considered uncomfortable or unnecessary by workers themselves [32,33,34,35].

Despite these well-documented hazards, many informal welders are unaware of relevant regulatory frameworks. In South Africa, the Occupational Health and Safety Act No. 85 of 1993 mandates that employers, including self-employed individuals, must provide and maintain a safe work environment. However, enforcement is weak in informal sectors, resulting in persistent safety violations and minimal accountability [6]. The lack of safety compliance at an institutional level not only endangers individual welders but also places surrounding communities at risk [7].

#### Supervision, Equipment Safety, and Site-Level Variability

The site-level heatmap analysis revealed considerable variation in safety compliance across informal welding workplaces. While a few sites demonstrated partial adherence to key safety measures, such as PPE availability, hygiene protocols, or ventilation, lacked even the most fundamental safety provisions, including fire extinguishers, first aid kits, or designated supervisors. This inconsistency reflects the broader ad hoc nature of informal work environments, where occupational health practices are often determined by individual site dynamics rather than formal standards.

Supervisory mechanisms were generally weak or entirely absent. In several cases, welding sites operated without any designated safety officer or formal oversight. However, in locations where informal peer-to-peer supervision existed, participants reported higher levels of compliance and better risk awareness. This suggests that even informal supervisory structures can influence behaviour positively when they promote safety norms through mentorship and mutual accountability.

The lack of standardized equipment and supervision has been widely documented in studies across the informal sector in sub-Saharan Africa. In Ghana, for instance, Atinga and colleagues [27] observed that informal welders operated with outdated or improvised tools, often shared among workers without clear maintenance protocols. Similarly, a study from Ethiopia found that unstructured supervision was a critical factor in persistent safety violations and contributed to the normalization of risk-taking behaviour [33].

### 4.2. Safety Awareness, Knowledge, and Reporting Behaviours

The study revealed that many welders have limited knowledge of occupational hazards and rarely report incidents due to fear of job loss, lack of access to medical facilities, or normalizing of injury within the workplace culture. More than half of the participants reported that they had never received formal safety training. This trend reflects broader evidence from LMICs, where training opportunities are scarce, especially for informal workers [8,9].

Furthermore, the reluctance to report injuries or seek treatment is compounded by economic vulnerability and the informal nature of employment. In Tanzania, for instance, informal welders often underreport injuries due to concerns over income disruption [28]. A similar pattern was observed in this study, where most welders expressed concerns that missing work due to injuries would directly affect their livelihoods.

### 4.3. Challenges Faced by Informal Welders

Informal welders in Pretoria West face a wide array of challenges that span physical, economic, and regulatory dimensions. Participants reported frequent exposure to physically demanding tasks, client pressure, faulty equipment, and unsafe work conditions. Welding was often performed at heights or in confined spaces, elevating the risk of burns, electric shocks, falls, and musculoskeletal injuries. These risks are intensified by unstable employment, the absence of contracts, and limited regulatory oversight [32,33].

Many participants expressed fear of specific hazards. One welder shared, *“Sometimes we don’t wear gloves because they are expensive”* (Male, age 45), while another added, *“I burned my fingers because I didn’t have proper gloves, only plastic ones”* (Male, age 32). These experiences reflect the intersection between poverty and safety non-compliance, echoing findings from informal welding settings in Durban and Johannesburg [2,33].

Most participants had acquired their skills through informal apprenticeships rather than structured education, leading to limited awareness of occupational safety measures. Similar patterns have been documented in many countries including, Nigeria, Indonesia, Ethiopia and Uganda [32,33,34,35,36,37,38,39], where welders demonstrated poor PPE usage and minimal occupational health knowledge. Many also reported chronic health issues, including fatigue, headaches, eye strain, and back pain. *“I experience constant headaches while welding,”* noted a participant (Male, age 29), while another reported, *“Welding has affected my eyes badly”* (Male, age 36).

These findings align with prior studies in Nepal [7], where prolonged exposure to welding fumes and inadequate ergonomic practices led to respiratory illnesses, skin conditions, and musculoskeletal disorders [35]. In Lahore, Pakistan, although 98.6% of informal welders reported access to PPE, most relied on substandard equipment and continued to experience high rates of arc eye injuries, burns, and back pain [37]. Awareness of chronic risks, such as metal fume fever and hearing loss, remained particularly low. This parallels our study, in which PPE availability and understanding were inadequate despite widespread risk exposure.

The regulatory vacuum surrounding informal welders in Pretoria West further exacerbates their vulnerability. Most participants lacked access to occupational health laws and operated without any formal documentation, permits, or contracts. A related study in Nepal, [7] similarly found that limited knowledge of OHS legislation contributed to poor compliance, indicating systemic enforcement failures. One study [38] underscored the value of procedural qualification systems in enhancing safety standards, suggesting that digital tools and standardized software could offer cost-effective solutions for informal welding contexts in South Africa.

This study fulfilled its objective of assessing PPE use, safety awareness, and hazard exposure among informal welders. Although a high proportion of participants were aware of basic safety practices, actual adherence was often limited due to comfort concerns, equipment affordability, and limited accountability structures. For example, a study in Ghana’s Jaman North District found that although 94.7% of welders claimed safety knowledge, many still used inadequate substitutes such as sunglasses instead of certified goggles [39]. Likewise, in Pretoria West, participants cited discomfort and inconvenience as reasons for poor PPE compliance, despite understanding the associated risks.

This gap between awareness and action is a recurring theme across informal economies, where apprenticeship-based learning dominates, and regulatory literacy remains low. The findings from both the survey and observational components of this study confirm that while welders often recognize the dangers of their work, meaningful implementation of safety practices remains inadequate.

### 4.4. Barriers to Healthcare Access and Legal Protection

Access to healthcare emerged as a major barrier for informal welders, particularly those who are undocumented or migrants. Participants cited a range of limitations, including the high cost of medical services, long distances to clinics, and fear of deportation or legal scrutiny. These challenges often led to underreporting of injuries and a reluctance to seek medical care, resulting in untreated conditions that may worsen over time.

This lack of access is a significant public health concern and reflects a broader systemic failure to protect vulnerable workers. Many participants were unaware of their legal entitlement to occupational health services, underscoring a critical knowledge gap in labor rights and protections. The situation stands in stark contrast to the goals of Sustainable Development Goal (SDG) 3, which emphasizes universal access to quality healthcare and decent working conditions [40].

A culture of fear and job insecurity also discouraged injury reporting. Several welders expressed concern that disclosing injuries could lead to job loss or retaliation, especially given the informal and unregulated nature of their employment. These fears limit the feedback loop necessary for workplace safety improvements. Similar barriers have been observed in studies from other low- and middle-income countries, where workers avoided disclosing health problems due to fear of dismissal or being misunderstood by employers [41,42].

In many of the sites observed, a safe and trusting work environment was notably absent. This lack of psychological safety conflicts with international best practices in occupational health and safety (OHS), which recommend supportive environments that encourage open reporting and timely responses to injuries. As highlighted by OHS management models, psychological and procedural trust are key components of effective hazard mitigation [43].

### 4.5. Comparative Literature Review: Situating Findings in South Africa and LMICs

Findings from this study are consistent with other studies, locally and internationally, where informal welders reported low PPE use and high injury rates due to regulatory gaps [32,33,34,35,36,37,38,39]. In these studies, welders lacked awareness of the Occupational Health and Safety Act No. 85 of 1993, similar to the majority of participants in Pretoria West.

Globally, parallels can be drawn with LMICs like Uganda, Ghana, Nepal,, and Indonesia. In these settings, informal sector workers also face inadequate access to training, PPE, and government protection [7,26,27,32]. In Ghana, for example, informal metal workers reported delays in seeking medical attention and limited safety inspections, similar to the Pretoria West context.

The lack of standardization and occupational risk findings also echo current research, where weak regulatory presence and lack of on-site safety officers were associated with increased injury prevalence [32,33,34,35,36,37,38,39]. Without consistent oversight, informal workers often prioritize productivity over precaution, especially when working under tight economic constraints or client pressure.

The results from this study emphasize the urgent need for structured, community-driven oversight mechanisms that can be integrated into informal settings. While formal inspections may be impractical, especially in mobile or unregistered operations, establishing peer-led safety roles, regular tool assessments, and low-cost certification programs may help introduce a baseline of compliance. Such initiatives have shown promise in pilot programs across informal sectors in the globe, where decentralized but structured safety promotion improved hazard awareness and reduced injury rates [44].

The international relevance of these findings underscores the need for global attention to informal worker safety in rapidly urbanizing regions.

### 4.6. Policy Implications for Informal Sector Occupational Health in South Africa

The findings of this study point to critical gaps in occupational safety compliance and protection for informal welders, necessitating a multidimensional policy response. At the municipal and Department of Health (DoH) level, there is a need to establish mobile occupational health and safety (OHS) outreach services specifically targeting informal sector workers. These mobile units can provide on-site inspections, distribute educational materials, and deliver basic health screenings. Additionally, the development of subsidies or the provision of low-cost PPE distribution programs would help alleviate economic barriers to safety compliance, enabling welders to access essential protective equipment.

There is also strong potential for integration with non-governmental organizations (NGOs) and non-profit organizations (NPOs) already working within informal employment sectors. These partnerships could be leveraged to implement short training modules and peer-led workshops on the correct use of PPE, hazard identification, and emergency response. Such initiatives would be particularly impactful if tailored to the context and literacy levels of the target communities.

Lastly, policy adjustments should consider the unique characteristics of informal workplaces. Enforcement strategies must be adapted for unregistered or mobile welding operations, which often fall outside the formal regulatory net. Safety education programs should also be simplified and delivered in accessible formats, accommodating workers with limited formal education. Together, these interventions could strengthen occupational health protections and promote safer working environments across South Africa’s informal welding sector.

### 4.7. Limitations

While this study provides valuable insights into occupational safety among informal welders, several limitations should be acknowledged. First, the sample was limited to 40 participants from Pretoria West, which constrains the generalizability of findings to broader populations of informal welders across South Africa. Additionally, the localized geographic scope may not capture variations in safety practices found in other urban or rural contexts.

Second, data collection relied in part on self-reported responses concerning injury history, PPE use, and awareness of occupational hazards. This approach may introduce recall bias and social desirability bias, with participants potentially underreporting injuries or overreporting safety behaviors.

Third, the observational component was constrained to specific sites and time frames, potentially limiting the ability to fully capture the variability and complexity of informal work environments. Informal welding operations often fluctuate in terms of personnel, location, and structure, making it difficult to standardize site-level comparisons.

Despite these limitations, the study employed a mixed-methods approach incorporating surveys, semi-structured interviews, and site observations, which enhanced the validity of the findings through triangulation. Future research with larger, more diverse samples across multiple regions would provide a more comprehensive understanding of occupational health dynamics in the informal welding sector.

### 4.8. Author’s Position on the Current Situation of Informal Welders in Pretoria West

The current situation of informal welders in Pretoria West reflects a widespread pattern as observed in other regions of the Global South, where safety awareness often fails to translate into safe practices. To address the identified challenges, several concrete and feasible measures are proposed:

- First, mandatory safety training programs should be rolled out targeting informal welders, and advising them to undergo training on sparks, fire risks, ventilation, PPE, and emergency response.
- Second, PPE distribution schemes subsidized or financed through public-private partnerships are critical, as affordability remains a core barrier. Various studies emphasized flexible payment schemes and government-subsidized PPE provision to promote safety adoption in low-income contexts.
- Third, legislative inclusion of informal welders into national occupational safety laws is essential. Currently, most safety regulations apply only to the formal sector. Pakistan’s experience highlights the urgent need to pass new legislation for the informal welding sectors and appoint qualified inspectors with the power to enter, assess, and improve working conditions.
- Fourth, community-level safety audits and licensing systems for informal workshops should be introduced to certify basic compliance with fire safety, protective equipment, and emergency preparedness standards. Finally, successful digital welding procedure qualification systems could be adapted for low-resource environments to guide welding practices and standardize quality and safety across informal operations. Implementing such context-specific, multi-level strategies could significantly improve occupational safety outcomes for informal welders in South Africa and beyond.

Therefore, the findings of this study have direct implications for the SDGs, particularly SDG 8, which promotes “decent work for all” and sustained economic growth. Informal welders in Pretoria West often operate under hazardous, unregulated conditions with limited access to PPE, formal training, or social protection realities that stand in stark contrast to the objectives of SDG 8. While informal welding enterprises provide essential livelihoods in South Africa’s urban townships, they remain excluded from labor protections afforded to the formal sector. This exclusion creates a dual burden of economic insecurity and elevated occupational risk.

To address this disparity, the study proposes a set of targeted interventions: expanding access to occupational health services, enforcing minimum safety standards, and developing incentive-based pathways to formalization. Such pathways may include voluntary registration schemes, tax incentives, subsidized PPE programs, and credits for safety training participation. These measures would not only improve working conditions for informal welders but also contribute directly to the achievement of Target 8.3 of SDG 8, which calls for “development-oriented policies that support productive activities, decent job creation, entrepreneurship, and the formalisation and growth of micro-, small-, and medium-sized enterprise”.

## 5. Conclusions

This study investigated the occupational safety risks and challenges faced by informal welders in Pretoria West, South Africa. Using a mixed-methods approach, it uncovered substantial gaps in safety knowledge, protective equipment use, training, and regulatory awareness. Findings revealed that welders frequently operate in hazardous environments with limited or no access to proper PPE, adequate ventilation, or functional equipment. Injuries such as burns, eye damage, and cuts were commonly reported, often linked to poor safety compliance and a lack of formal training.

Qualitative insights further illustrated the complex pressures these workers face, including demanding client expectations, economic constraints, and limited healthcare access, especially among undocumented foreign welders. Many welders learn their trade informally and remain unaware of their rights under national safety legislation such as the Occupational Health and Safety Act No. 85 of 1993.

These findings echo concerns raised in studies from other LMICs, including Uganda, Tanzania, Ghana, and Ethiopia, reinforcing the global nature of occupational vulnerability in informal sectors. They also align with South African research in cities like Johannesburg and Durban, which identified similar gaps in enforcement, training, and PPE accessibility among informal workers.

The study confirms that informal welders in Pretoria West operate under hazardous conditions with limited safety oversight, with the most common occupational risks and injuries, which include burning, getting an electric shock, and cutting themselves as they perform their tasks. This requires an urgent need for practical interventions such as expanding access to safety training, promoting affordable PPE, and adapting regulatory strategies to fit informal workplaces. Strengthening partnerships with NGOs, community organizations, and local municipalities can offer a pathway to safer, more sustainable informal work environments, and government bodies should consider implementing vocational training programs, PPE subsidies, and periodic safety inspections. Future research should expand to other regions and include larger samples to build a more comprehensive national picture of informal occupational safety.

## Figures and Tables

**Table 1:** Welders’ demographics (age).

Age Intervals	Number (n)	Percentage (%)
20–30	18	45%
31–40	13	32.5%
41–50	7	17.5%
51–60	2	5%
TOTAL =	40	100%

**Table 2:** Occupational information (type of welding) of the participants included in the study.

Type of Welding	Number (n)	Percentage (%)
Arc welding	4	10%
Gas welding	34	85%
MIG welding	2	5%
TOTAL =	40	100%

**Table 3:** Barriers to healthcare reporting and comfort in discussing health concerns with the employer.

Variables	Number of Participants
**1. Barriers to Healthcare or Reporting Work-Related Injuries/Illnesses:**	
a. No Significant Barriers	14 (35%)
b. Related to Foreigner Status/Lack of Passport	6 (15%)
c. Financial Barriers	2 (5%)
d. Logistical Barriers (Distance/Time Off)	2 (5%)
e. Reliance on Self-Treatment	2 (5%)
f. Unspecified Barriers	14 (35%)
**2. Comfort Level Discussing Health Concerns with Employer:**	
a. Not Comfortable	24 (60%)
b. Comfortable	9 (22.5%)
c. No Employer (Self-Employed)	7 (17.5%)

## Data Availability

No new data were created.

## Abbreviations

The following abbreviations are used in this manuscript:

Abbreviation	Full Term
OHS	Occupational Health and Safety
PPE	Personal Protective Equipment
LMICs	Low- and Middle-Income Countries
DoH	Department of Health
NGO	Non-Governmental Organization
NPO	Non-Profit Organization
SDG	Sustainable Development Goal
SA	South Africa

## References

[1] Min W., Lin G.S. (2019). Manufacture of Biomaterials. Encycl. Biomed. Eng..

[2] Magoha L., Nyanza E.C., Asori M., Thomas D.S.K. (2024). Informal Welders’ Occurred Safety and Environmental Health Risks in North-Western Tanzania. Glob. Public Health.

[3] International Association of Drilling Contractors (IADC) (2020). Welding: Confined Space. https://iadc.org/safety-meeting-topics/welding-confined-space/.

[4] Mrema E.J., Ngowi A.V., Mamuya S.H.D. (2015). Status of Occupational Health and Safety and Related Challenges in the Expanding Economy of Tanzania. Ann. Glob. Health.

[5] Chiboyiwa E., Ncube F., Erick P. (2022). Risk Factors for Work-Related Musculoskeletal Disorders Among Welders in the Informal Sector Under Resource-Constrained Settings. Work.

[6] Republic of South Africa. 1993. Occupational Health and Safety Act, No. 85 of 1993. Government Gazette 14918, 23 June 1993. https://www.gov.za/documents/occupational-health-and-safety-act.

[7] Budhathoki S.S., Singh S.B., Sagtani R.A., Niraula S.R., Pokharel P.K. (2014). Awareness of Occupational Hazards and Use of Safety Measures Among Welders: A Cross-Sectional Study from Eastern Nepal. BMJ Open.

[8] Maharja J., Faradisha A.M.F., Panggeleng A. (2022). Safety Behaviour Tendency Among Welders by Utilising Personal Protective Equipment. Public Health Sci. J..

[9] Creswell J.W. (2014). Research Design: Qualitative, Quantitative, and Mixed Methods Approaches.

[10] Schoonenboom J. (2023). The Fundamental Difference Between Qualitative and Quantitative Data in Mixed Methods Research. Forum Qual. Sozialforschung Forum Qual. Soc. Res..

[11] Bryman A. (2016). Social Research Methods.

[12] Tashakkori A., Teddlie C. (2010). SAGE Handbook of Mixed Methods in Social & Behavioral Research.

[13] Statistics South Africa (2011). Census 2022.

[14] Makwana D., Engineer P., Dabhi A., Chudasama H. (2023). Sampling Methods in Research: A Review. Int. J. Trend Sci. Res. Dev..

[15] Abubakar A.M., Gubbio B.S., Musa A. (2025). Assessment of the Awareness of Occupational Hazards and Practice of Safety Measures Among Welders in Maiduguri Metropolis, Borno State, Nigeria. J. Afr. Adv. Sustain. Stud..

[16] Microsoft Corporation (2016). Microsoft Excel. https://office.microsoft.com/excel.

[17] Miles M.B., Huberman A.M. (1994). Qualitative Data Analysis: An Expanded Sourcebook.

[18] Bryman A., Cramer D. (2011). Quantitative Data Analysis with IBM SPSS 17, 18 and 19: A Guide for Social Scientists.

[19] Patton M.Q. (2015). Qualitative Research and Evaluation Methods.

[20] Braun V., Clarke V. (2006). Using Thematic Analysis in Psychology. Qual. Res. Psychol..

[21] Busetto L., Wick W., Gumbinger C. (2020). How to Use and Assess Qualitative Research Methods. Neurol. Res. Pract..

[22] GraphPad Software (2025). Quantify Interrater Agreement with Kappa. https://www.graphpad.com/quickcalcs/kappa1/.

[23] Republic of South Africa Protection of Personal Information Act 4 of 2013. Government Gazette 37067, 26 November 2013. https://www.gov.za/documents/protection-personal-information-act/.

[24] Alli B.O. (2002). Fundamental Principles of Occupational Health and Safety.

[25] Mushi L. (2019). Factors Affecting Adherence to Occupational Health and Safety Rules and Regulations in the Informal Sector. Int. J. Ind. Psychol..

[26] Itiakorit B., Zziwa E.B., Osuret J. (2021). Prevalence and Determinants of Occupational Injuries Among Welders in Small-Scale Metal Workshops in Wakiso District, Uganda. East Afr. Health Res. J..

[27] Murugan S.S., Sathiya P. (2024). Analysis of Welding Hazards from an Occupational Safety Perspective. Minist. Sci. Technol. Vietnam.

[28] Nalugya A., Kiguli J., Wafula S.T., Nuwematsiko R., Mugambe R.K., Oputan P., Tigaiza A., Isunju J.B., Ssekamatte T. (2022). Knowledge, Attitude and Practices Related to PPE Among Welders in Uganda. Health Psychol. Behav. Med..

[29] Department of Labour Regulations for Hazardous Work on Machinery. Government Gazette No. 25207, 14 July 2003. https://www.gov.za/sites/default/files/gcis_document/201409/25207a0.pdf.

[30] (2009). Health and Safety in Welding and Allied Processes.

[31] International Labour Organization (2013). An Introduction to Occupational Health and Safety.

[32] Oluwole I., Nwanna K., Afolabi K.K., Ademola S.A., Aremu A.B., Mujeeb S. (2018). Determinants of Compliance with Occupational Health Practices Among Welders in Uganda. Med. Saf. Glob. Health.

[33] Afework A., Tamene A., Tafa A. (2024). Compliance with OHS Measures Among Small-Scale Metal Workers in Ethiopia. Risk Manag. Healthc. Policy.

[34] Obarhoro O.I., Nwufo C.R. (2020). Compliance in the Use of PPE by Welders in Delta State, Nigeria. Int. J. Res. Rev..

[35] Nastiti A., Prabaharyaka I., Roosmini D., Kunaefi T.D. (2012). Health-Associated Cost of Urban Informal Sector. Procedia Soc. Behav. Sci..

[36] Hassan S.M., Nasir U., Anwar K., Talib U. (2017). PPE Use and Hazard Awareness Among Welders in Pakistan. Int. J. Occup. Environ. Health.

[37] Mayur B.W., Pratibha W. (2020). Occupational Hazards Associated with Welding Work. Int. J. Curr. Res. Rev..

[38] Wang R., Jianying H., Mingju M. (2020). Design and Implementation of Welding Qualification Expert System. J. Phys. Conf. Ser..

[39] Eric E., Martha D. (2018). Wayside Welders’ Safety Measures in Welding: A Case of Jaman North. University of Education, Winneba. http://ir.uew.edu.gh:8080/handle/123456789/4503.

[40] United Nations (2015). Transforming Our World: The 2030 Agenda for Sustainable Development.

[41] Balkhyour M.A., Ahmad I., Rehan M. (2019). PPE Use and Occupational Exposures in Small Industries. Saudi J. Biol. Sci..

[42] Reason J. (1997). Managing the Risks of Organizational Accidents.

[43] Tadesse S., Bezabih K., Destaw B., Assefa Y. (2016). Awareness of Occupational Hazards Among Welders in Ethiopia. J. Occup. Med. Toxicol..

[44] Man A.B.C. (1993). Safety and Health in the Use of Chemicals at Work: A Training Manual.

